# Using multiwinner voting to search for movies

**DOI:** 10.1007/s11238-024-10012-0

**Published:** 2024-11-15

**Authors:** Grzegorz Gawron, Piotr Faliszewski

**Affiliations:** 1 AGH University and VirtusLab, Kraków, Poland; 2 AGH University, Kraków, Poland

**Keywords:** Multiwinner voting, Recommendation system, Information retrieval, MovieLens

## Abstract

We show a prototype of a system that uses multiwinner voting to suggest resources (such as movies) related to a given query set (such as a movie that one enjoys). Depending on the voting rule used, the system can either provide resources very closely related to the query set or a broader spectrum of options. We show how this ability can be interpreted as a way of controlling the diversity of the results. We test our system both on synthetic data and on the real-life collection of movie ratings from the MovieLens dataset. We also present a visual comparison of the search results corresponding to selected diversity levels.

## Introduction

The idea of multiwinner voting is to provide a committee of candidates based on the preferences of the voters. In principle, such mechanisms have many applications, ranging from choosing parliaments, through selecting finalists of competitions, to suggesting items in Internet stores or services; see, e.g., the discussions provided by Elkind et al. (2017b) and Faliszewski et al. (2017c), and the overview of Lackner and Skowron (2023). While the first two types of applications indeed are quite common in practice, the last one, so far, was viewed mostly as a theoretical possibility. Our goal is to change this view. To this end, we design a prototype of a voting-based search system that given a movie (or, a set of movies), finds related ones. The crucial element of our system—enabled by the use of multiwinner voting—is that one may specify to what extent he or she wants to focus on movies very tightly related to the given one, and to what extent he or she wants to explore a broader spectrum of movies that are related in some less obvious ways. Indeed, if someone is looking for movies exactly like the specified one, then using focused search is natural. However, if someone is not really sure what he or she really seeks, or if he or she has already watched the most related movies, looking at a broader spectrum is more desirable.

Viewed more formally, our system belongs to the class of non-personalized recommendation systems based on collaborative filtering. That is, from our point of view the users posing queries are anonymous and we do not target the results toward particular individuals, but rather we try to find movies related to the ones they ask about. In this sense, we provide more of a search-support tool than a classical recommendation system.

To find the relationships between the movies, we use a dataset of movie ratings (in our case, the MovieLens dataset of Harper and Konstan 2016). Such a dataset consists of a set of agents who rate the movies on a scale between one and five stars, where one is the lowest score and five is the highest. For each movie we consider which agents enjoyed it and what other movies these agents liked. More specifically, given the raw data with movie ratings we form a global election where we indicate which users liked which movies. To this end, we say that a user liked a movie if he or she gave it at least four stars; in the language of voting literature, liking a movie corresponds to *approving* it. Then, given a query, i.e., either a single movie or a set of movies, we restrict this election to the agents who liked the movies from the query and to the movies that these agents liked, except for the ones from the query. Based on this local election, for each user and each movie that he or she likes, we determine a utility score which indicates how relevant the movie is (briefly put, we need to distinguish between movies that are globally very popular, such as, e.g., *The Lord of the Rings*, from the ones that are mostly popular among the agents in the local election). Finally, we seek a winning committee with respect to one of the OWA-based multiwinner voting rules discussed by Skowron and Faliszewski (2016), and output its contents as our result [see also the works of Aziz et al. (2017) and Bredereck et al. (2020) for further discussions of these rules]. Since, in general, our rules are ${\textrm{NP}}$-hard to compute (Skowron & Faliszewski, 2016; Aziz et al., 2015), we use approximation algorithms and heuristics.

OWA-based rules are parameterized by the *ordered weighted average* operators of Yager (1988) and, depending on the choice of these operators, they may provide committees of very similar, individually excellent candidates, or of more diverse ones. This is illustrated in the simulations of Elkind et al. (2017a); Faliszewski et al. (2017b), and Godziszewski et al. (2021), and explained theoretically by Aziz et al. (2017) and Lackner and Skowron (2020, 2021). Thus, by choosing the OWA operators appropriately, we either find movies very closely related to a given query, or those that form a broader spectrum of related movies. Specifically, we use a family of operators parameterized by a value $p \ge 0$, such that for $p=0$ we get the most focused results, and for larger *p*’s the results become more broad.

**Our contribution.** Our main contribution is designing a voting-based search system and testing it in the context of selecting movies. In particular, we show the following results:

1. Using movie-inspired synthetic data, we show that, indeed, the rules that are meant to choose closely related movies or more broad committees, do so. Using the MovieLens dataset, we observe that the system provides appealing results on sample queries.
2. We use our system to visualize relations between movies. On the one hand, this allows us to observe that our voting rules act as intended also on real-life data. On the other hand, it provides insight into the nature of the movies.

While our system is a prototype, we believe that its results are promising and deserve further study.

**Related work.** Regarding multiwinner voting, we point the readers to the overview of Faliszewski et al. (2017c), who discuss various multiwinner voting rules and their possible applications, and to the book of Lackner and Skowron (2023), who focus on approval-based rules. It is also interesting to consider the work of Elkind et al. (2017b), where the idea of using multiwinner voting for selecting movies was suggested, albeit in a somewhat different setting, and to the survey of Rey and Maly (2023), who discuss participatory budgeting (which is a form of multiwinner voting where candidates have possibly different costs and we seek committees whose total cost does not exceed a given budget). While multiwinner voting is not yet a mainstream tool in applications, several researchers have used it successfully. For example, Chakraborty et al. (2019) have shown that appropriate multiwinner rules can be used to select trending topics on Twitter or popular news on the Internet. For the latter task, Mondal et al. (2020) also designed a voting-based solution. Pourghanbar et al. (2015) and Faliszewski et al. (2017a) used diversity-oriented multiwinner voting rules to design genetic algorithms that would avoid getting stuck in plateau regions of the search space. After the conference version of our paper, Streviniotis and Chalkiadakis (2022) also applied multiwinner voting in a recommender system, but for the case of a tourism domain and in a somewhat different way than us.

For a broad discussion of modern recommendation systems, see the handbook edited by Ricci et al. (2015), and, for an early account of collaborative filtering methods, see the work of Sarwar et al. (2001). As examples of works on movie recommendations, we mention (a) the paper of Ghosh et al. (1999), who described a movie recommendation system using Black’s voting rule with weighted user preferences, (b) the paper of Azaria et al. (2013), who focused on maximizing the revenue of the recommender, (c) the paper of Choi et al. (2012), who discussed recommendations based on movie genres, and (d) the paper of Phonexay et al. (2018), who adapted some techniques from social networks to recommendation systems. Most of this literature aims at finding the most tightly related movies and, as such, is less relevant to our study. There are works on recommendation systems that focus on diversifying the results, such as, e.g., the work of Kim et al. (2019), but in this paper the authors use neural networks and rely on a number of features, whereas we use multiwinner voting and simple collaborative filtering. Further, they do not focus on movies so it is difficult to compare the results. Information bubble is defined by Nikolov et al. (2015) as a filter limiting user’s access to certain results. Our approach gives the users full control of the query being executed, as well as the diversity level of the results they require. They can always opt for highest level of diversity reflecting as large number of voters’ opinions as possible, so they have a chance of escaping from the filter bubble.

Let us review notions of diversity that appear in the context of voting, recommendation systems, and information retrieval. Drosou et al. (2017) pointed out that diversity is an ambiguous notion and is understood differently, depending on the context. In this paper, we are committed to diversity understood as aggregated dissimilarity of the resources in the winning committee. This viewpoint is similar to the one presented by Celis et al. (2016), who define *combinatorial diversity* to mean the entropy of the distribution of candidate features in the result set and *geometric diversity* to relate to the volume of a hyper cube with vertices being the space-embedded candidates. However, we do not use any of the candidates’ features, relying instead on the user preferences only. Indeed, this position is closely related to the view of diversity in multiwinner elections, as discussed by Faliszewski et al. (2017c), where a diverse winning committee is expected to represent as many voters as possible. Formally, this can be understood as maximizing the number of voters that approve at least one member of the committee. This approach is similar to the concept of representativeness described by Chasalow and Levy (2021), where the problem is to select a subsample that *represents* a larger population. Celis et al. (2018) additionally sought committees whose members are diverse in terms of additional features—such as, e.g., gender or seniority level.

Next we discuss other notions of diversity, which are orthogonal to our approach, but whose knowledge helps in understanding our scope. Clarke et al. (2008) described diversity as a tool to respond to user’s potential multiple intents when using an ambiguous query. This is different from our approach because we do not expect the user to have some specific intent that we do not know, but rather that the user is not sure him or herself what the intent truly is. Clarke et al. (2008) also defined a related concept of *novelty* to avoid returning duplicate items if present, but this does not apply to our setting as we provide non-personalised search, which does not keep track of the history of the queries. Mitchell et al. (2020) referred to the variety in the result set with respect to any potential candidate characteristic as *heterogeneity*, while reserving the term *diversity* to variety with respect to purely sociopolitical characteristics, such as gender, age, or race. Finally, there is a number of measures of diversity used in the information retrieval context, but they do not fit our setting (Järvelin & Kekäläinen, 2002). Indeed, all of them depend on a defined order of results or/and defined query intents.

## Preliminaries

Let ${\mathbb {R}}_+$ denote the set of nonnegative real numbers, and for a positive integer *i*, let [*i*] denote the set $\{1, \ldots , i\}$.

**Utility and approval elections.** Let $R = \{r_1, \ldots , r_m\}$ be a set of *resources* and let $N = \{1, \ldots , n\}$ be a set of *agents* (in other papers, the resources are often referred to as the *candidates* and the agents are often referred to as the *voters*). Each agent *i* has a utility function $u_i :R \rightarrow {\mathbb {R}}_+$, which specifies how much he or she appreciates each resource. We assume that the utilities are comparable among the agents and that the utility of zero means that an agent is completely uninterested in a given resource. We do not normalize utility values, so, for example, some agent may be far more excited about the resources than some other one. Committees are sets of resources, typically of a given size *k*. For a committee $S = \{s_1, \ldots , s_k\}$ and an agent *i*, by $u_i(S)$ we mean the vector $(u_i(s_1), \ldots , u_i(s_t))$, where the utilities appear in some fixed order over the resources; however, this order will never be relevant for our discussion. We write $U = (u_1, \ldots , u_n)$ to denote a collection of utility functions, referred to as a *utility profile*. A *utility election* $E = (R,U)$ consists of a set of resources and a utility profile over these resources, where the utility profile also implicitly specifies the set of agents. An *approval election* is a utility election where each utility is either 1, meaning that an agent approves a resource, or 0, meaning that he or she does not approve it. For approval elections we typically denote the utility profile as $A = (a_1, \ldots , a_n)$ and call it an *approval profile*. For a resource $r_t$, we write $A(r_t)$ to denote the set of agents that approve it.

**OWA operators.** An *ordered weighted average* (OWA) operator is specified by a vector of nonnegative real numbers, such as $\lambda = (\lambda _1, \ldots , \lambda _k)$, and works as follows. For a vector $x = (x_1, \ldots , x_k) \in {\mathbb {R}}^k$ and a vector $x' = (x'_1, \ldots , x'_k)$ obtained by sorting *x* in the nonincreasing order, we have:

1 $$\begin{aligned} \lambda (x) = \lambda _1 x'_1 + \lambda _2 x'_2 + \cdots + \lambda _k x'_k. \end{aligned}$$

For example, operator $(1, \ldots , 1)$ means summing up the elements of the input vector, whereas operator $(1,0,\ldots , 0)$ means taking its maximum element. OWA operators were introduced by Yager (1988).

**(OWA-based) multiwinner voting rules.** A *multiwinner voting rule* is a function *f* which, given a utility election *E* and an integer *k*, returns a family of size-*k* winning committees. We focus on OWA-based rules.

Consider a utility election $E = (R,U)$, where $R = \{r_1, \ldots , r_m\}$ and $U = (u_1, \ldots , u_n)$, and an OWA operator $\lambda = (\lambda _1, \ldots , \lambda _k)$. Let *S* be a size-*k* committee. We define the $\lambda$-score of committee *S* in election *E* to be $\lambda{\mathrm{-}}score_E(S) = \sum _{i=1}^{n} \lambda (u_i(S))$. We say that a multiwinner rule *f* is OWA-based if there is a family $\Lambda = (\lambda ^{(k)})_{k \ge 1}$ of OWA operators, one for each committee size *k*, such that for each election *E* and each committee size *k*, *f*(*E*, *k*) consists exactly of those size-*k* committees *S* for which $\lambda ^{(k)}{\mathrm{-}}score_E(S)$ is highest.

**HUV rules.** We are particularly interested in the rules that use OWA operators of the followig form, where $p \ge 0$:

2 $$\begin{aligned} \lambda ^p = (1, \nicefrac {1}{2^p}, \nicefrac {1}{3^p}, \ldots ) \end{aligned}$$

and we refer to them as *p*-*Harmonic Utility Voting* rules (*p*-HUV rules). The name stems from the fact that for $p = 1$, their OWA operators sum up to harmonic numbers. Let us consider three special cases:

1. For a committee size *k*, the 0-HUV rule chooses *k* resources with the highest total utility; indeed, its OWA operator is $(1, \ldots , 1)$. Under approval elections, 0-HUV is the classic *Multiwinner Approval Voting* rule (AV).
2. The 1-HUV rule uses OWA operators $(1, \nicefrac {1}{2}, \nicefrac {1}{3}, \ldots )$; for approval elections this is the *Proportional Approval Voting* rule (PAV) of Thiele (1895).
3. Abusing the notation, $\infty$-HUV is a rule that uses OWA operators $(1,0, \ldots , 0)$; for approval elections it is the *Chamberlin–Courant* rule (CC); originally introduced by Chamberlin and Courant (1983) for the ordinal setting and converted to the approval one by Procaccia et al. (2008) and Betzler et al. (2013).

In the approval voting setting, these three rules correspond to the three main principles of choosing committees. AV chooses *individually excellent* resources, i.e., those that are appreciated by the largest number of agents; PAV chooses committees that, in a certain formal sense, proportionally represent the preferences of the agents (Aziz et al., 2017; Brill et al., 2018), and CC ($\infty$-HUV) focuses on *diversity*, i.e., it seeks a committee so that as many agents as possible appreciate at least one item in the committee. For a more detailed description of these principles, see the overview of Faliszewski et al. (2017c). For a focus on approval rules, see the book of Lackner and Skowron (2023) and their work on the opposition between AV and CC (Lackner & Skowron, 2020).

We proceed under two premises. The first one is that the 0-HUV, 1-HUV, and $\infty$-HUV rules extend the principles of individual excellence, proportionality, and diversity to the setting of utility elections (the visualizations of Elkind et al. (2017a) and Godziszewski et al. (2021) support this view). The second one is that for $p > 1$, the rule *p*-HUV provides committees that achieve various levels of compromise between those of 1-HUV and $\infty$-HUV (this is supported by the results of Faliszewski et al. (2017b). We do not consider *p* values between 0 and 1).

**Computing HUV committees.** Unfortunately, for each $p > 0$ it is ${\textrm{NP}}$-hard to tell if there is a committee with at least a given score under the *p*-HUV rule (Skowron & Faliszewski, 2016; Aziz et al., 2015) and, as a consequence, no polynomial-time algorithms are known for these rules (for 0-HUV it suffices to sort the candidates in terms of their total utilities and, up to tie-breaking, take top *k* ones). We consider two ways of circumventing this issue:

1. We use the standard greedy algorithm: To compute a *p*-HUV committee of size-*k* (for some $p > 0$), we start with an empty committee and perform *k* iterations, where in each iteration we extend the committee with a single resource that maximizes its *p*-HUV score. A classic result on submodular optimization shows that the committees computed this way are guaranteed to achieve at least $1 - {1}/{e} \approx 0.63$ fraction of the highest possible score (Nemhauser et al., 1978).
2. We use the simulated annealing heuristic (Kirkpatrick et al., 1983), as implemented in the *simanneal* library, version 0.5.0. We set the number of steps to 50,000 and the temperature to vary between 9900 and 0.6. Briefly put, simulated annealing proceeds as follows: First, we sample a random committee. Then, we perform a number of iterations where in each of them we replace a random committee member and check if this improves the committee’s score. If so, then we keep the new committee. If not, then we revert the change with some probability that depends on the current temperature value; the higher the temperature, the higher the chance of accepting a lower-scoring committee. As the iterations progress, the tempareture keeps decreasing (the idea is that accepting a locally worse solution allows simulated annealing to jump out of a local optimum and find a global one). For more details, see, e.g., the overview of Delahaye et al. (2019).

In principle, we could have used the formulations of *p*-HUV rules as integer linear programs (ILPs), provided, e.g., by Skowron and Faliszewski (2016) and Peters and Lackner (2020). Yet, given the sizes of our elections this would be quite infeasible (e.g., for the movie *Alice in Wonderland (1951)* we obtain an election with 32,783 resources and 5339 agents). Further, in addition to simulated annealing we also could have used a number of other metaheuristics, such as, e.g., various forms of ant collony optimization (Dorigo & Stützle, 2019). We did not pursue this direction as our heuristics already give satisfying outcomes (as also confirmed by the results of Faliszewski et al. 2018).

## System design

Let us now describe our voting-based search system. The main idea is that for a *query set* of resources—such as a set of movies that someone enjoys—we form an election that regards related resources and whose winning committee is our *result*. Depending on the voting rule used, the result may contain resources either very closely or only somewhat loosely connected to those from the query. The system consists of three main components, the data model, the search model, and winner determination.

### Data model

The data model is responsible for converting domain-specific raw data into what we call a *global (approval) election*. For example, the raw data may consist of information how various people rate movies, or what products they buy in some store, or it may be generated using some statistical model of preferences. This last approach is particulary useful to test our system in a controlled environment.

The global election stores our full knowledge of the domain. The interpretation is that the agents are the users who have interacted with some resources and they approve those for which the interaction was positive; for example, if they enjoyed a particular movie. Lack of an approval either means that the interaction was negative or that there was no interaction. While we could distinguish these two cases, we find using basic approval elections to be simpler.

The algorithm for forming the global election is the only domain-specific part of our model. Below we provide an example of how such an algorithm may work.

#### Example 1

Consider the MovieLens 25 M dataset (Harper & Konstan, 2016). It contains 25,000,095 ratings of 62,423 movies, provided by 162,541 users (so, on average, each user rated almost 154 movies). Each rating is on the scale from one to five stars and was provided between January of 1995 and November of 2019 on the MovieLens website. The more stars an agent assigned to a movie, the more this agent liked it. We form a global election where each user is an agent, each movie is a resource, and a user approves a movie if he or she gave it at least four stars. We remove from consideration those movies that were approved by fewer than 20 agents.

### Search model

The search model is responsible for forming a *local (utility) election*, specific to a particular query. The idea is that this election’s winning committees would form our result sets. We first form a local approval election and then, if desired, we derive more fine-grained utilities for the agents.

Let $E = (R,A)$ be the global approval election, where $A = (a_1, \ldots , a_n)$, and let $Q \subseteq R$ be the query set. Let $N = \{1, \ldots , n\}$ be the set of agents present in *E* and let $N_{ local }$ be a subset of *N* containing those agents who approve at least one member of *Q*.^1^ Then, let $R_{ local }$ consist of those resources that are approved by the agents from $N_{ local }$, except those from the query:

3 $$\begin{aligned} R_{ local }= \{ r \in R \mid (\exists i \in N_{ local })[ a_i(r) = 1]\} \setminus Q. \end{aligned}$$

Finally, let $A_{ local }$ be the approval profile of the agents from $N_{ local }$, restricted to the resources from $R_{ local }$, and let $E_{ local }= (R_{ local }, A_{ local })$ be our local approval election. Intuitively, it contains the knowledge about exactly those resources that were appealing to (some of) the agents that also enjoyed members of *Q*. Unfortunately, as shown below, it may be insufficient to provide relevant search results.

#### Example 2

Consider the MovieLens global election from Example 1 and let the query set *Q* consist of a single movie, *Hot Shots!*, a 1991 parody of the *Top Gun (1986)* movie, full of quirky/absurd humor. The five most-approved movies in the local approval election for *Q* are:

(1) *The Matrix (1999)*,

(2) *Back to the Future (1985)*,

(3) *Fight Club (1999)*,

(4) *Pulp Fiction (1994)*, and

(5) *Lord of the Rings: The Fellowship of the Ring (2001).*

This is also the winning committee under the AV rule with $k=5$. Neither of these movies has much to do with *Hot Shots!*, and they were selected because they are globally very popular. Indeed, we expect many more readers of this paper to have heard of these five movies than of the one from the query set. Such globally popular movies are also popular among people enjoying *Hot Shots!.*

To address the above issue, we derive a local utility election $E_{{ util }}= (R_{{ util }},U_{{ util }})$, where $R_{{ util }}= R_{ local }$, which promotes those resources that are more particular to a given query. To this end, we use the *term frequency-inverse document frequency (TF-IDF)* mechanism.

**TF-IDF.** This is a standard heuristic introduced by Jones (1973, 2004) to evaluate how specific is a given term *t* for a document *d* from a document corpus *D* [(for further information on TF-IDF see, e.g., the works of Robertson and Walker (1997) and Ounis (2018)]. The main idea is that the specificity value of *t* in *d* is proportional to the frequency of *t* in *d* (term frequency; *TF*) and inversely proportional to the frequency of *t* in all the documents *D* (inverse document frequency; *IDF*).

Given our global election $E = (R,A)$ and the approval local election $E_{ local }= (R_{ local },A_{ local })$, we implement the TF-IDF idea as follows. We interpret the resources as the terms, and we take the document corpus to consist of two “documents,” election $E_{ local }$ and election $E' = (R_{ local },A')$, where $A'$ is the approval profile for those agents from the global election that do not appear in $E_{ local }$. Let *n* be the total number of agents. For a resource $r \in R_{ local }$, we let its *term frequency* component be the number of agents that approve it in the local election, i.e., $tf (r) = |A_{ local }(r)|.$ We let *r*’s inverse document frequency be $idf (r) = \ln \left( \nicefrac {n}{|A(r)|} \right) .$ Finally, to balance the TF and IDF components, we assume we have some constant $\gamma$ and we define:

4 $$\begin{aligned} tf \hbox {-} idf _\gamma (r) = tf (r) \gamma ^{ idf (r)} = \textstyle \frac{|A_{ local }(r)|}{|A(r)|^{\ln \gamma }} \cdot \left( n^{\ln \gamma } \right) . \end{aligned}$$

#### Example 3

Consider three resources, $r_1$, $r_2$, and $r_3$, where: $|A_{ local }(r_1)| = 1$, $|A(r_1)| = 2$, $|A_{ local }(r_2)| = 10$, $|A(r_2)| = 20$, and $|A_{ local }(r_3)| = 100$, $|A(r_3)| = 2000$. If we focused on the number of approvals in the local election (by taking $\ln \gamma = 0$), then we would view $r_3$ as the most relevant resource. This would be unintuitive as only a small fraction of $r_3$’s approvals come from the agents who enjoy the items in the query set. For $\gamma \approx 2.78$ (i.e., $\ln \gamma = 1$), we would focus on the ratios $\nicefrac {|A_{ local }(r_i)|}{|A(r_i)|}$, so $r_1$ and $r_2$ would be equally relevant, and $r_3$ would come third. This is more appealing, but still unsatisfying as, generally, $r_2$ is more popular than $r_1$. By taking, e.g., $\gamma = 2$ (i.e., $\ln \gamma \approx 0.69$) we would focus on ratios $\nicefrac {|A_{ local }(r_i)|}{|A(r_i)|^{0.69}}$ and, indeed, $r_2$ would be the most relevant resource, followed by $r_1$ and $r_3$.

We have found that $\gamma = 1.85$ works best for our scenario (we discuss the process of choosing this value in Sect. 4.1).

#### Example 4

Consider the same setting as in Example 2, but take five movies with the highest TF-IDF value (for $\gamma = 1.85$). We obtain: (1) *The Naked Gun 2 1/2 (1991)*, (2) *Hot Shots! Part Deux (1993)*, (3) *Top Secret! (1984)*, (4) *The Naked Gun (1988)*, (5) *The Loaded Weapon 1 (1993)*. All these movies are parodies similar in style to *Hot Shots!*.

**Local utility election.** We form the local utility election $E_{{ util }}= (R_{{ util }},U_{{ util }})$ by setting the utilities as follows. Given an agent *i* from the local approval election and a resource $r \in R_{{ util }}$, if agent *i* approves *r*, then we set $u_i(r) = \nicefrac { tf \hbox {-} idf (r)}{|A_{ local }(r)|}$. Otherwise, we set it to be 0. This way the utilities assigned to a given resource sum up to its TF-IDF value.

#### Example 5

By the design of the local utility election, a 0-HUV committee of size $k=5$ for the *Hot Shots!* local utility election would consist exactly of the five movies listed in Example 4.

### Winner determination

The last component of our system is to compute (an approximation of) a winning committee under the local utility election under a given *p*-HUV rule. If we are looking for resources that are the most closely connected to the query set, then we take $p = 0$. For a broader search, we consider $p \in \{1,2,3, \ldots \}$. Since most of our rules are ${\textrm{NP}}$-hard to compute (Skowron & Faliszewski, 2016; Aziz et al., 2015), we either use the greedy approximation algorithm or simulated annealing. The greedy algorithm returns the committee ordered with respect to the iteration number in which a given resource was added (thus, the first resource is always the same for a given election, irrespective of *p*). The algorithm based on simulated annealing outputs the committee in an arbitrary order.

**Table 1:** Results provided by our system for the movie *Hot Shots!* (see Examples 1, 4, and 6)

	Exact algorithm	Simulated annealing		Greedy algorithm	
#	0-HUV	1-HUV	2-HUV	1-HUV	2-HUV
1	The Naked Gun 2 1/2 (1991)	Hot Shots! Part Deux (1993)	Hot Shots! Part Deux (1993)	The Naked Gun 2 1/2 (1991)	The Naked Gun 2 1/2 (1991)
2	Hot Shots! Part Deux (1993)	The Loaded Weapon 1 (1993)	The Loaded Weapon 1 (1993)	Hot Shots! Part Deux (1993)	The Loaded Weapon 1 (1993)
3	Top Secret (1984)	The Naked Gun 2 1/2 (1991)	The Villain (1979)	The Loaded Weapon 1 (1993)	Major League II (1994)
4	The Naked Gun (1988)	Cannonball Run II (1984)	Top Secret (1984)	Major League II (1994)	Yamakasi (2001)
5	The Loaded Weapon 1 (1993)	Top Secret (1984)	Ernest Goes to Jail (1990)	Top Secret (1984)	Hot Shots! Part Deux (1993)
6	Police Academy (1984)	Nothing to Lose (1997)	Last Boy Scout, The (1991)	Yamakasi (2001)	To Be or Not to Be (1983)
7	The Last Boy Scout (1991)	Dragnet (1987)	Dragnet x(1987)	Hudson Hawk (1991)	Hudson Hawk (1991)
8	Commando (1985)	Major League II (1994)	Freaked (1993)	To Be or Not to Be (1983)	Freaked (1993)
9	Hudson Hawk (1991)	Yamakasi (2001)	Major League II (1994)	City of Violence (2006)	Top Secret (1984)
10	Twins (1988)	Coffee Town (2013)	Yamakasi (2001)	Dragnet (1987)	City of Violence (2006)

#### Example 6

Consider the local utility election for the *Hot Shots!* movie. In Table 1 we show the *p*-HUV committees for, $p \in \{0,1,2\}$, where for 0-HUV we use the exact algorithm and for the other two rules we use simulated annealing and the greedy algorithm. Let us discuss the contents of these committees (for $p \in \{1,2\}$, we focus on simulated annealing):

1. The first six movies selected by 0-HUV are quirky, absurd comedies, quite in spirit of *Hot Shots!*. Among the next four movies, three are comedies (one of which is somewhat similar in spirit to the first six) and one is an action movie.
2. Except for *Yamakasi*, all movies selected by 1-HUV are comedies of different styles, including four of the same style as *Hot Shots!*, two action comedies, two family comedies, and one crime comedy. *Yamakasi* is an action/drama movie which stands out from the rest.
3. 2-HUV selects even more varied set of comedies than 1-HUV, including a western comedy, a sci-fi comedy, crime/action comedies, and family movies. Yet, it also includes *Yamakasi*.

The reason why *Yamakasi* is included in our 1-HUV and 2-HUV committees is simply because, in total, it only received 53 approvals, of which 27 came from people who enjoyed *Hot Shots!*. Thus it was viewed as a very relevant movie for the query. If we replaced the simple TF-IDF heuristic with a more involved scoring system (possibly using more information about the movies), we could account for such situations better.

The committees computed by the greedy algorithm for 1-HUV and 2-HUV are of comparable quality to those provided by simulated annealing, although they include eight comedies and two action movies each.

## Experiments

In this section we present a number of experiments, mostly conducted on the MovieLens dataset. In the first experiment (Sect. 4.1), we describe our process of choosing the $\gamma$ value for the TF-IDF heuristic. In the second one (Sect. 4.2), conducted on synthetic data, we test if, indeed, 0-HUV rule focuses on resources very similar to the one from the query set, whereas *p*-HUV rules for $p \in \{1,2,3\}$ seek increasingly more diverse result sets (we often omit the results for $p=3$ as they are similar to those for $p=2$). In the third experiment (Sect. 4.3) our goal is similar as in the second one, but we analyse real-life data and we present a certain visualization of the search results. Finally, in Sect. 4.4 we present the measurements of committee diversity averaged across 1000 search results for movies from the Movielens dataset.

### Calibrating the TF-IDF metric

The main purpose of the first experiment is to choose the value of the $\gamma$ parameter for the TF-IDF metric in a principled way.

**Fig. 1:**
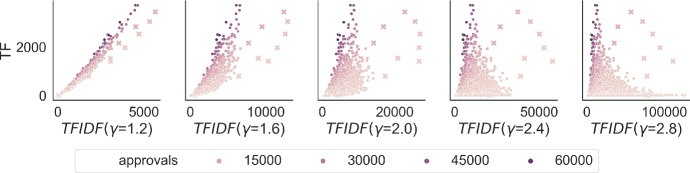
StarTrek calibration details. Each dot (or cross) represents a movie in the local election generated for *Star Trek III: The Search for Spock (1984)* and shows the relation between its popularity (its *TF* value) on the *y* axis and its final TF-IDF score on the *x* axis, for various values of $\gamma$. Crosses represent other movies from the Star Trek series. The hue of the dot (or cross) represents the number of its approvals in the global election

Intuitively, $\gamma$ is used to give more weight to the IDF component relative to the TF one. In other words, replacing $\gamma$ with a larger value more strongly diminishes the TF-IDF values of the globally more popular movies than the less popular ones. This balance is visualised in Fig. 1, where we consider the movie *Star Trek III: The Search for Spock (1984)* as a singleton query, and for each movie in the local approval election we draw a dot (or a cross, if it is another *Star Trek* movie) whose *y* coordinate is its number of approvals in the local election (i.e., its TF value) and whose *x* coordinate is its TF-IDF value, for several values of $\gamma$. The hue of the dot/cross represents the number of approvals of the movie in the global election. The top movies according to TF-IDF (for a given $\gamma$) are the rightmost dots/crosses in the respective diagram. Note that the higher the $\gamma$ is, the more dots/crosses with low TF value appear to the right. For $\gamma =1.2$, quite a few generally popular items (with darker hue) make it to the top, simply because they are popular overall and not only in the context of the query. For $\gamma =2.0$, there seems to be a good balance between the popular and not so popular movies, while for $\gamma =2.8$ there are only unpopular movies in the top.

The above argument for using $\gamma = 2.0$ is based on intuition and, indeed, it turns out that a slightly different value gives somewhat better results. To find this value we have performed the following experiment. Our basic premise is that the $\gamma$ value should be such that when searching for a singleton query, its most similar movies should appear among the top ten with respect to the TF-IDF metric. While deciding what is “the most similar movie” is subjective, we identified certain clusters of movies which are similar to one another within the same cluster. For example, for movies from the *Star Trek* series, other *Star Trek* movies should be viewed as the most similar ones. The MovieLens dataset contains fourteen *Star Trek* movies (that are approved by at least 20 users). We list the *Star Trek* movies present in the MovieLens dataset, together with their approval counts in the global election, in Table 2a.

**Table 2:** The list of the Star Trek movies from MovieLens 25 M dataset (left) used in the calibration experiment and the results of calibrating the TF-IDF heuristic (right) showing the relation between $\gamma$ and the average number of Star Trek movies found in the top 10 results according to TF-IDF when searching for each of the Star Trek movies

Movie	Approval Count
*(a) Star Trek movies present in the MovieLens 25 M dataset (with at least 20 approvals), together with their approval counts*	
Star Trek: Renegades	27
Star Trek: Nemesis	1904
Star Trek Beyond	1987
Star Trek V: The Final Frontier	2338
Star Trek: Insurrection	3783
Star Trek: The Motion Picture	3785
Star Trek III: The Search for Spock	4732
Star Trek VI: The Undiscovered Country	5358
Star Trek Into Darkness	5621
Star Trek IV: The Voyage Home	7544
Star Trek II: The Wrath of Khan	10,669
Star Trek: Generations	10,809
Star Trek: First Contact	12,396
Star Trek	12,854

$\gamma$	Average top ten count
*(b) Star Trek TF-IDF selected calibration results*	
1.65	6.71
1.7	6.93
1.75	6.93
1.8	7.0
1.85	7.14
1.9	**7**.**21**
1.95	**7**.**21**
2.0	**7**.**21**
2.05	7.0
2.1	6.93
2.15	6.93
2.2	6.93
2.25	6.64
2.3	6.36

**Fig. 2:**
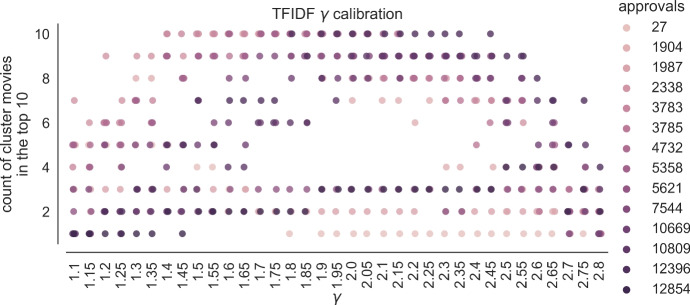
The Star Trek calibration experiment results. Each dot represents the *top 10 count* value (vertical axis) when searching for a single movie (hue represents the popularity of the movie, the darker the more approvals the movie has) using TF-IDF with a given $\gamma$ (horizontal axis)

We used each *Star Trek* movie as a singleton query set, computed its local approval election, and ranked the movies from this election with respect to their TF-IDF values for $\gamma$ between 1.10 and 2.80 (with a step of 0.05). We show the results of these computations in Fig. 2. The interpretation of this figure is as follows. For each value of $\gamma \in \{1.1, 1.15, \ldots , 2.80\}$, we present all the 14 *Star Trek* movies as dots. The *y* coordinate of each dot is the number of other *Star Trek* movies that are among top-ten ones when we use the given movie as a singleton query set. The colour of each dot corresponds to the number of approvals it receives in the global election. In Table 2b we present the average *y* coordinates of the dots from Fig. 2, averaged over all *Star Trek* movies, for each of our values of $\gamma$. We obtain the highest result for $\gamma \in \{1.9, 1.95, 2.0\}$.

**Fig. 3:**
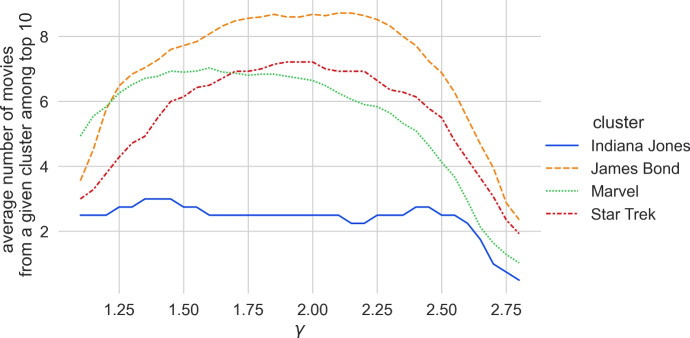
Calibration graph for all the separately considered movie clusters. For each of the considered clusters we show the average count of the cluster movies in the top 10 results when searching for one of the movies from the same cluster for various values of $\gamma$

**Fig. 4:**
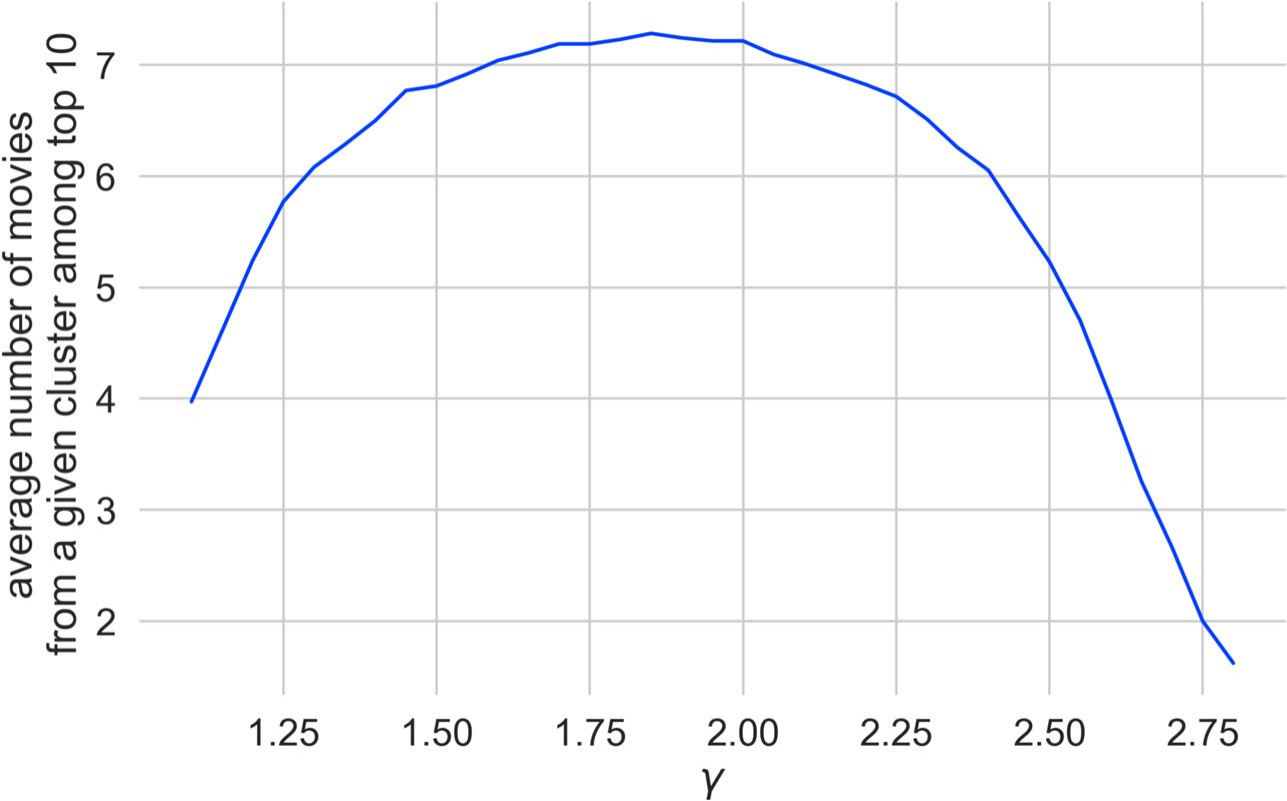
A joint calibration graph, averaging the results from the cluster of *Star Trek*, *Indiana Jones*, *James Bond*, and *Marvel* movies

However, we found that for other such clusters we obtain different $\gamma$ values. We checked four movies from the *Indiana Jones* series (and obtained the highest results for $\gamma \in \{1.35, 1.40, 1.45\}$), 25 movies from the *James Bond* series (and obtained the highest result for $\gamma = 1.85$) and 31 movies from the *Marvel* universe (and obtained the highest result for $\gamma = 1.6$). Figure 3 shows the calibration graphs for all these distinct clusters. Since there is not a single optimal $\gamma$ value for all clusters, we decided to use the average of the top 10 movie counts across all the movies from the mentioned clusters. The results are presented in Fig. 4. We fix the $\gamma = 1.85$, for which we obtained the highest average.

### Testing the rules: synthetic data

In the second experiment, we generate the global election synthetically. The point is to observe the differences between committees computed according to *p*-HUV rules for different values of *p* in a controlled environment.

**Fig. 5:**
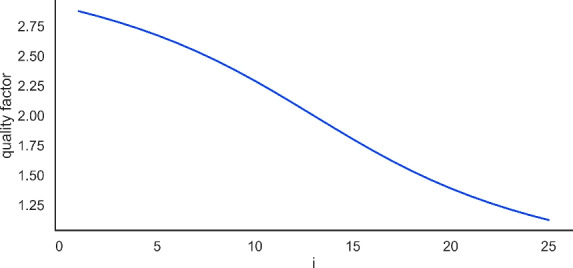
The *quality factor* function which defines the relationship between the quality of a synthetic movie and its index

**Generating global elections.** We assume that we have nine main categories of movies (such as, e.g., a comedy or a thriller) and each category has nine subcategories (such as, e.g., a romantic comedy, or a psychological thriller). For each pair of a category $u \in \{1, \ldots , 9\}$ and its subcategory $v \in \{1, \ldots , 9\}$, we generate 25 movies, denoted $u.v(1), \ldots , u.v(25)$. Given a movie $u.v(i)$, we set its *quality factor* to be $q(i) = -\arctan {\frac{i-13}{10}}+2$. That is, for each subcategory the first movie has the highest quality, about 2.87, and the qualities of the following movies decrease fairly linearly, down to about 1.12 (naturally, this choice is quite arbitrary; we plot the quality function in Fig. 5).

We have $n = 2000$ voters. Each voter *i* has a probability distribution $P_i$ over the main categories and for each category *u*, he or she has probability distribution $P_i^{u}$ over its subcategories. For each voter, we choose each of these distributions as permutations of (0.5, 0.1, 0.1, 0.1, 0.1, 0.025, 0.025, 0.025, 0.025) , chosen uniformly at random: Each voter has the most preferred category, four categories that he or she also quite enjoys, and four categories that he or she rarely enjoys (the same applies to subcategories). To generate an approval of a voter *i* we do as follows: (1) We choose a category *u* according to distribution $P_i$ and, then, a subcategory *v* according to distribution $P_i^u$. (2) We choose a movie among $u.v(1), \ldots , u.v(25)$ with probability proportional to its quality factor. The voter approves the selected movie. We repeat this process 162 times for each voter, leading to a bit fewer approvals due to repetitions in sampling (recall that in MovieLens the average number of approvals is 154). While this process is certainly quite ad-hoc, we believe that it captures the main features of preferences regarding movies. Further, in this model two movies are very similar if they come from the same subcategory, are somewhat similar if they come from the same category but different subcategories, and are very loosely related otherwise.

**Fig. 6:**
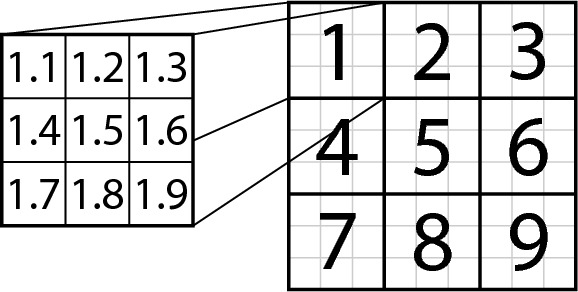
Visual arrangement of the movie categories and subcategories for the synthetic experiment

**Running the experiment.** For each number $p \in \{0, 1, 2, 3\}$ and both algorithms for computing approximate *p*-HUV committee, we repeat the following experiment (we do not show results for $p=3$ as they are similar to those for $p=2$). For each committee size $k \in \{5,10,15,20\}$ we generate 100 global elections as described above^2^ and for each of them we compute a winning committee of a given size, for the query set consisting of movie $1.1(13)$, i.e., the middle-quality movie from subcategory 1.1 (since the (sub)categories are symmetric, their choice is irrelevant; results for other movies are similar). Considering all runs of the experiment, altogether $100\cdot k$ movies are selected (some are selected more than once and we count each occurrence separately). Then, for each subcategory *u*.*v*, we sum up how many movies from this subcategory are among the selected movies, obtaining a histogram.

**Fig. 7:**
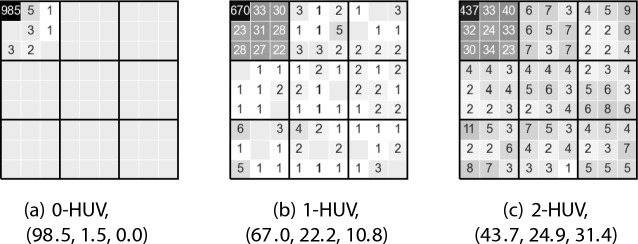
Histograms for the synthetic experiment and the greedy algorithm ($k=10$)

**Fig. 8:**
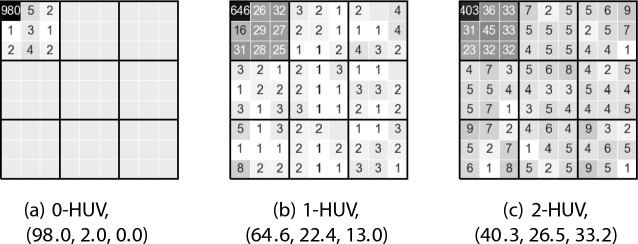
Histograms for the synthetic experiment and simulated annealing ($k=10$)

To present these histograms visually, we arrange the categories into a $3 \times 3$ square, where each category is further represented as a $3 \times 3$ sub-square of subcategories, as shown in Fig. 6. We show thus-arranged histograms for the greedy algorithm in Fig. 7 and for simulated annealing in Fig. 8 (generally, they are quite similar). Each subcategory/square is labelled with the number of movies selected from this subcategory and its background reflects this number (darker backgrounds correspond to higher numbers). Further, next to the name of each *p*-HUV rule we report a vector (*x*, *y*, *z*), where *x* means the percentage of the movies selected from subcategory 1.1, *y* means the percentage of the movies selected from category 1 except for those in subcategory 1.1, and *z* refers to the percentage of all the other selected movies. Thus we always have that $x+y+z = 100$. Finally, in Tables 3 and  4 we report the (*x*, *y*, *z*) vectors achieved for both our algorithms and for different committee sizes.

**Table 3:** The (*x*, *y*, *z*) vectors achieved in the synthetic experiment for different rules and different committee sizes *k* using the greedy algorithm (numbers rounded to a single decimal)

*k*	Greedy algorithm		
	0-HUV	1-HUV	2-HUV
5	(99.8, 0.2, 0.0)	(83.4, 14.8, 1.8)	(65.8, 23.6, 10.6)
10	(98.5, 1.5, 0.0)	(67.0, 22.2, 10.8)	(43.7, 24.9, 31.4)
15	(96.5, 3.4, 0.1)	(57.7, 23.4, 18.9)	(35.2, 25.4, 39.4)
20	(92.4, 7.6, 0.1)	(52.6, 24.6, 22.9)	(30.5, 26.4, 43.1)

**Table 4:** The (*x*, *y*, *z*) vectors achieved in the synthetic experiment for different rules and different committee sizes *k* using simulated annealing (numbers rounded to a single decimal)

*k*	Simulated annealing		
	0-HUV	1-HUV	2-HUV
5	(99.6, 0.4, 0.0)	(83.2, 14.0, 2.8)	(62.2, 24.6, 13.2)
10	(98.0, 2.0, 0.0)	(64.6, 22.4, 13.0)	(40.3, 26.5, 33.2)
15	(94.6, 5.3, 0.1)	(55.5, 23.4, 21.1)	(31.3, 26.4, 42.3)
20	(88.4, 11.3, 0.4)	(49.3, 25.1, 25.6)	(27.8, 27.1, 45.1)

**Analysis.** Our main conclusion is that, indeed, 0-HUV focuses on very similar movies (almost all the selected movies come from category 1.1) and as *p* increases, approximate *p*-HUV committees include more and more movies from other subcategories of category 1, and, eventually, even more movies outside of it. It would be desirable to have a value of *p* for which we would get a vector (*x*, *y*, *z*) close to, say, (45.0, 45.0, 10), so that about half of the movies would be very related to the query, about half would be quite related, and few would be rather loosely related; meaning that, on average, the resulting committee of size $k=10$ would contain between 4 and 5 movies from category 1.1 (i.e., directly relevant to the search), between 4 and 5 movies from other subcategories of category 1 (i.e., similar but quite different from the query), and 1 movie from some other category (i.e., something very different, but possibly appealing to the people who enjoyed the movie from the query). Our algorithms do not seem to provide committees with such vectors and finding rules that would provide them would be interesting (this, however, is not a major worry—after all, the setup in the experiment was simplified and, to some extent, Example 6 shows that for real-life MovieLens data we do find such committees).

The second conclusion is that the results regarding the committees computed by the greedy algorithm and by simulated annealing are rather similar and a more careful statistical analysis would be needed to distinguish between them. We do not perform such more detailed analysis and leave it for future work.

Finally, we observe how the (*x*, *y*, *z*) vectors depend on the committee size *k* (see Tables 3, 4). For 0-HUV, the committee size has relatively little influence: Aside for $k=20$—which is fairly extreme as there are only 25 movies in each of the subcategories—the rule focuses on the most relevant movies, i.e., those from category 1.1, irrespective of the committee size. The situation for 1-HUV and 2-HUV is different. While in most cases the *y*-value in their vectors does not change much with the committee size (except for the case of $k=5$), the larger a committee we consider, the larger is the *z* component at the expense of the *x* one. That is, the rules select proportionally fewer directly relevant movies and choose more loosely connected ones. While, perhaps, it would be more appealing if the *y* component increased as well—or, even, instead of component *z*—overall this means that when the rules have more space to seek diverse committees, afforded by the larger committee size, the rules do take advantage of this, which is the main intent.

### Testing the rules: movies data

In this section we observe how varying the value of *p* affects the results of *p*-HUV rules on the MovieLens dataset, and how our system can find relations between the movies. Our strategy is as follows: First, we derive a (dis)similarity measure between the movies. Then, we gather a set of movies—including some that we want to focus on and some that appear as search results when we query for the former ones—and present them on a 2D plane, so that the more similar two movies are, the closer they are to each other. We annotate this visualization with the movies that we query for and the search results. This way we see how 0-HUV selects movies very similar to the query, while other rules choose more spread-around sets. Next, as a bit of an anecdotal example, we apply our dissimilarity measure to the clusters of movies that we used for TF-IDF calibration. For each of the clusters, our measure gives meaningful results, discovering not-completely-obvious phenomena (such as the fact that a given movie series had a reboot). This reinforces our belief that the measure is truly useful. In this section we focus on the greedy algorithm.

**(Dis)similarity among movies.** Let us consider two movies, *x* and *y*. We take the following approach to obtain a number that, in some way, is related to their similarity. First, we form a local utility election using *x* as the singleton query set. If *y* does not appear in this election, then we consider it as completely dissimilar from *x*. Otherwise, we sort the movies from the local election in the ascending order of their TF-IDF values and we define the rank of *y* with respect to *x*, denoted ${{\textrm{rank}}}_x(y)$, to be the position on which *y* appears. (So if *y* has the highest TF-IDF value then ${{\textrm{rank}}}_x(y) = 1$, if it has the second highest TF-IDF then ${{\textrm{rank}}}_y(x)= 2$, and so on; recall that the idea of TF-IDF is that the higher it is, the more relevant a movie is for the search query and, so, we equate relevance with similarity). We define the dissimilarity between *x* and *y* as ${{\textrm{diss}}}(x,y) = \nicefrac {1}{2}\big ( {{\textrm{rank}}}_x(y) + {{\textrm{rank}}}_y(x) \big ).$ This ensures that ${{\textrm{diss}}}(x,y) = {{\textrm{diss}}}(y,x)$ and that the larger ${{\textrm{diss}}}(x,y)$ is, the less related—and, hence, less similar—are the two movies.

Our dissimilarity measure has some flaws. For example, let us consider two sets of movies, *A* and *B*, where the movies within each of the sets are very similar to each other. Further, let us assume that *A* contains significantly more movies than *B*. Then the average dissimilarity between the movies in *A* would be considerably larger than the dissimilarity between the movies in *B*, even if objectively there would be no justification for such a difference. While we acknowledge that such issues may happen, we view them as somewhat extreme and we expect that in most situations our measure is sufficient to distinguish between movies that are clearly related and those that do not have much to do with each other.

**Gathering movies for comparison.** We would like to compare the outcomes of various *p*-HUV rules on each of the movies from some set *A*. To do so, we form an extension of *A* as follows (we consider *p* values in $\{0,1,2,3\}$ and committee size $k=10$, unless we say otherwise): (1) For each movie $x \in A$ and each $p \in \{0,1,2,3\}$, we compute the *p*-HUV winning committee for the local utility election based on the singleton query *x*. We take the union of these committees and call it *B*. (2) For each movie $y \in B$, we compute 0-HUV winning committee of size two for the local utility election based on *y*. We refer to the union of these committees as *C*. (3) We let ${{\textrm{ext}}}(A) = A \cup B \cup C$ be the extension of *A*. We use set *B* because we are interested in relations between the contents of the committees provided by all our rules for all the movies in *A*, and we add set *C* because we also want to ensure that each movie from $A \cup B$ has a similar one in the extension.

**Fig. 9:**
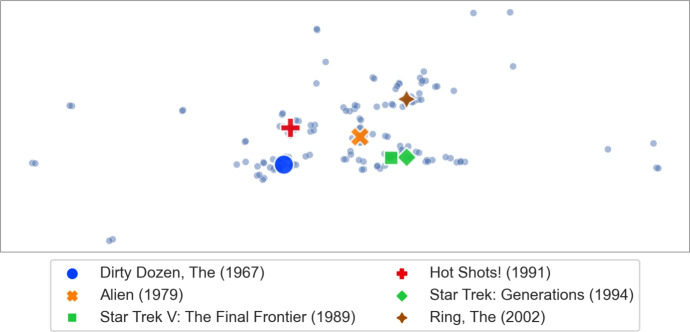
Embedding of several example movies, chosen to be diverse

**Visualizing relations between the movies.** Given a set *A* of movies and its extension ${{\textrm{ext}}}(A)$, we first compute the value ${{{\textrm{diss}}}}(x,y)$ for all distinct movies *x* and *y* in ${{\textrm{ext}}}(A)$. Then, we form a complete graph where members of ${{\textrm{ext}}}(A)$ are the nodes and for each two movies *x* and *y*, the edge connecting them has weight ${{{\textrm{diss}}}}(x,y)$. Then we compute an embedding that maps each movie in ${{\textrm{ext}}}(A)$ to a point on a two-dimensional plane, so that the Euclidean distances between these points correspond (approximately) to the weights of the edges. To this end, we use the force-directed algorithm of Fruchterman and Reingold (1991). We use the implementation provided in the *networkx* library (version 2.6.3), ran for 10’000 iterations. For a description of the library, we refer to the work of Hagberg et al. (2008).

The Fruchterman-Reingold algorithm does not take the weights of the edges in the graph whose embedding it is to compute as input, but the forces that act to bring the nodes of a given edge closer. Since this value should be inverse to our dissimilarity, for each two movies *x* and *y* we use force $({{{\textrm{diss}}}}(x,y))^{-2}$ (by experimenting with different force functions we found this value to work well).

**Comparing p-HUV rules.** Next we use the visualization methodology to analyse the outcomes of different *p*-HUV rules. Set *A* consists of movies *Hot Shots! (1991)*, *Star Trek V: The Final Frontier (1989)*, *Star Trek: Generations (1994)*, *Alien (1979)*, *Ring, The (2002)*, and *Dirty Dozen, The (1967)*. *Hot Shots!* is a quirky comedy, *Star Trek* movies are examples of science-fiction, and so is *Alien*, which also has strong elements of a horror movie. *Ring, The* is a horror movie and *Dirty Dozen, The* is a classic war movie. We show an embedding of these movies (and the movies from their extension) in Fig. 9. In particular, we see that the two *Star Trek* movies are correctly presented as very similar, whereas the other movies are farther away from each other.

**Fig. 10:**
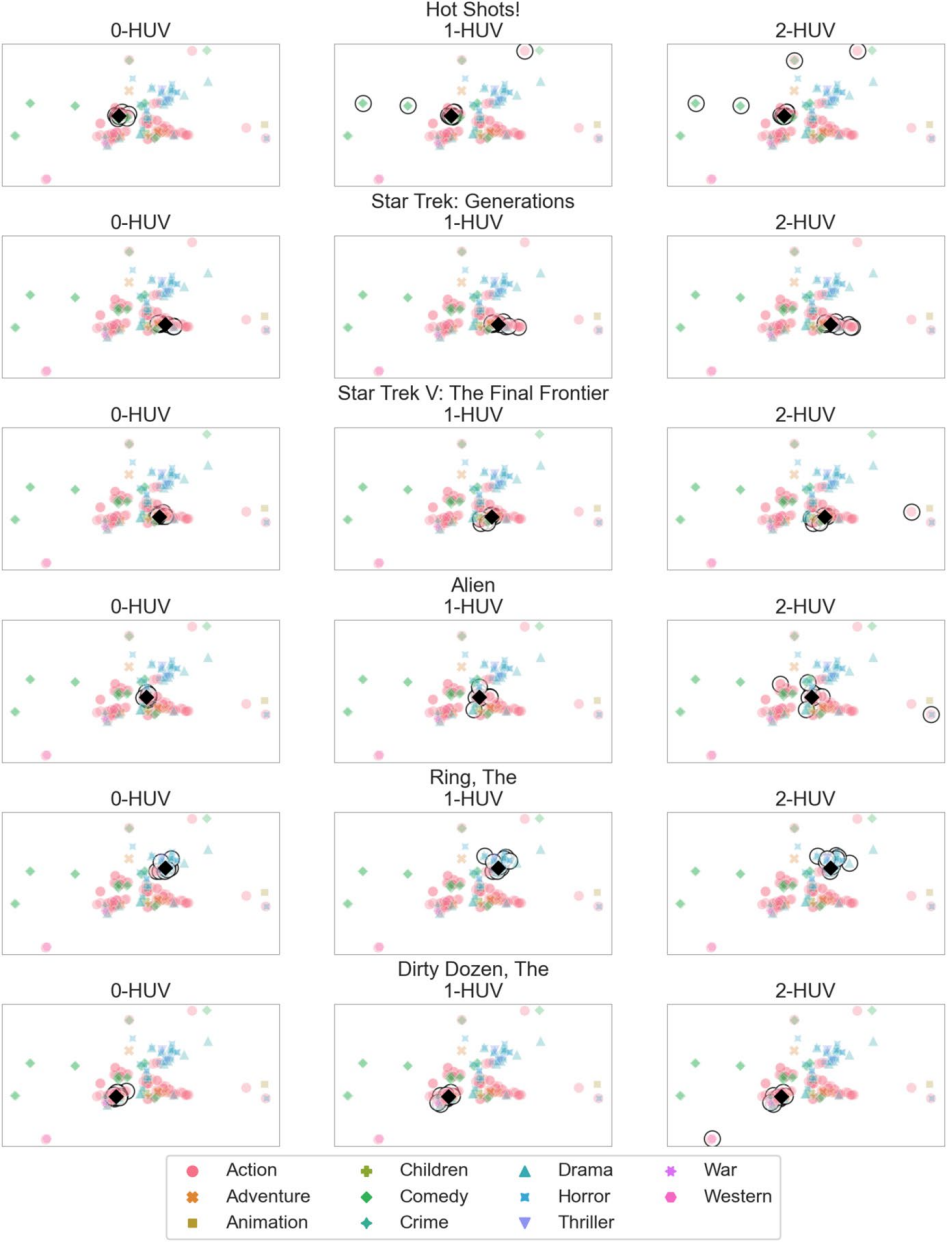
Search results for various queries and various *p*-HUV rules

In Fig. 10 we visualise the committees provided by *p*-HUV rules for $p \in \{0,1,2\}$ and singleton query sets from *A*. Specifically, each column corresponds to a value of *p* and each row to a different query. The query is marked with a black diamond and the members of selected committees have surrounding black circles (they are guaranteed to be present in the extension of *A*). Each movie is coloured with respect to the first genre provided for it in MovieLens (in this dataset each movie can have multiple genres, listed in some order).

We see that the 0-HUV rule always chooses movies very close (very similar) to the one from the query. Indeed, we have defined our dissimilarity function to encode this effect. It is more interesting to consider *p*-HUV rules for $p \ge 1$. In this case, we see that the selected movies are always farther away from the query than for 0-HUV, but the extent to which this happens varies. For example, for *Hot Shots!* the committees get more and more spread as *p* increases, whereas for *Star Trek: Generations* the outcomes are very similar for all $p \ge 1$. Yet, altogether, our system achieves its main goals: The 0-HUV rule gives tightly focused results, closely connected to the query, and *p*-HUV rules for $p \ge 1$ give more diverse results.

**Fig. 11:**
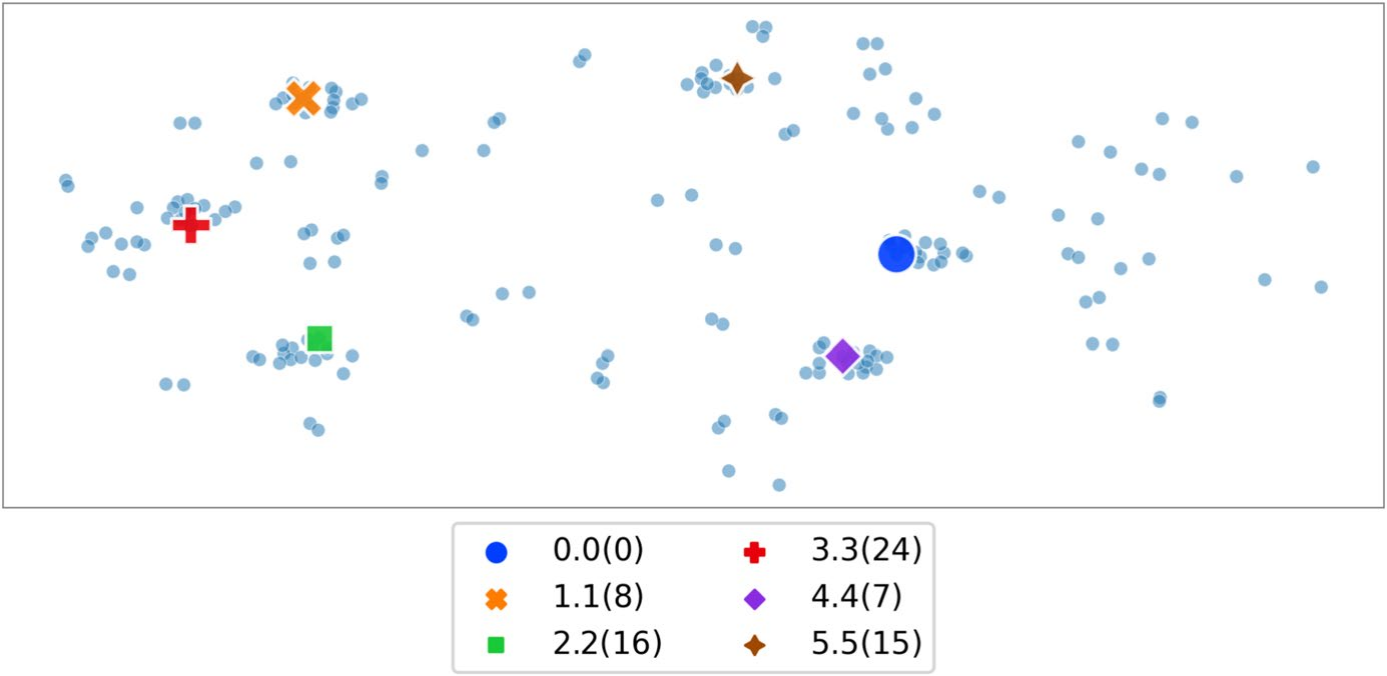
Embedding map of synthetic data

**Fig. 12:**
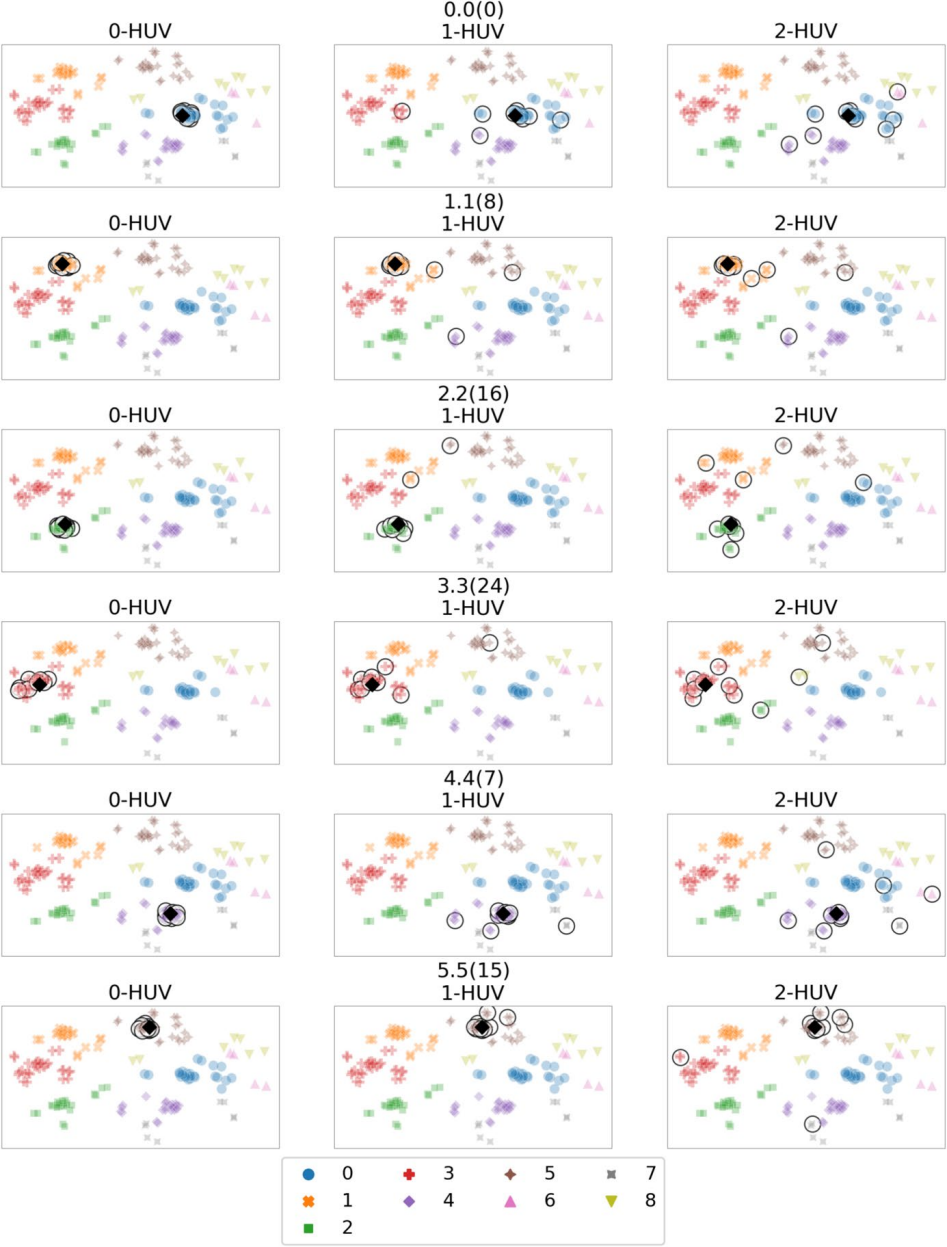
Synthetic data search results for various candidates and various *p*-HUV rules

Next, we repeat the just-described experiment, but using synthetic data from Sect. 4.2. To this end, we generate synthetic global election, we select various candidates from 6 main categories: 0.0(0), 1.1(8), 2.2(16), 3.3(24), 4.4(7), 5.5(15), we follow the extension procedure to obtain a bigger set of candidates, and, finally, we present the results in Figs. 11 and 12. The first observation is that the synthetic candidates, compared to the real-life movies, are more evenly spread on the map (Fig. 11) and their main categories are grouped in more isolated clusters, marked with different colours on Fig. 12; see also Fig. 10. This can be explained by more noise in real-life movies dataset, which results in noisy distribution of forces used by the Fruchterman-Reingold algorithm (Fruchterman & Reingold, 1991). The second observation is that, as expected, the 2-HUV results spread out to other main categories to a larger extent than 1-HUV and 0-HUV results.

**Fig. 13:**
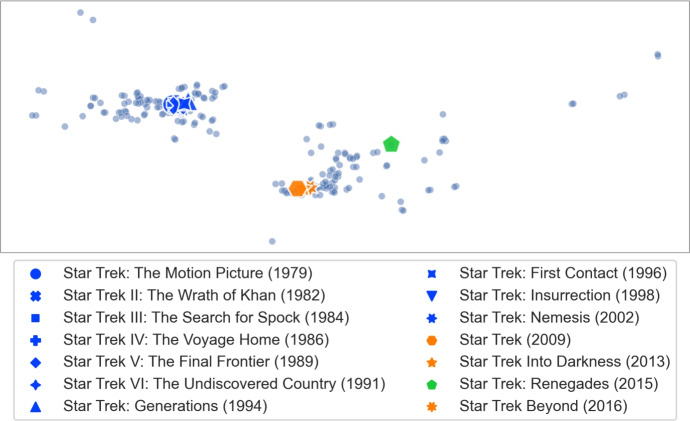
Embedding of Star Trek movies

**Fig. 14:**
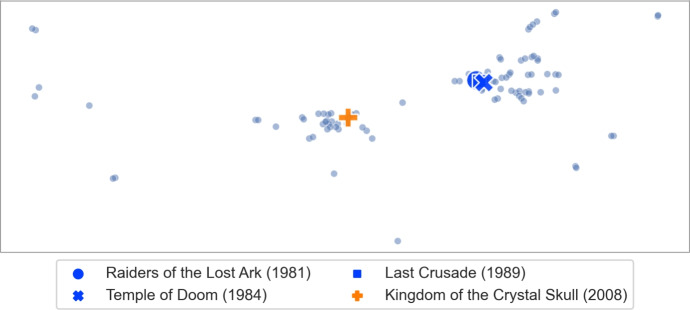
A embedding map of *Indiana Jones* movies

**Fig. 15:**
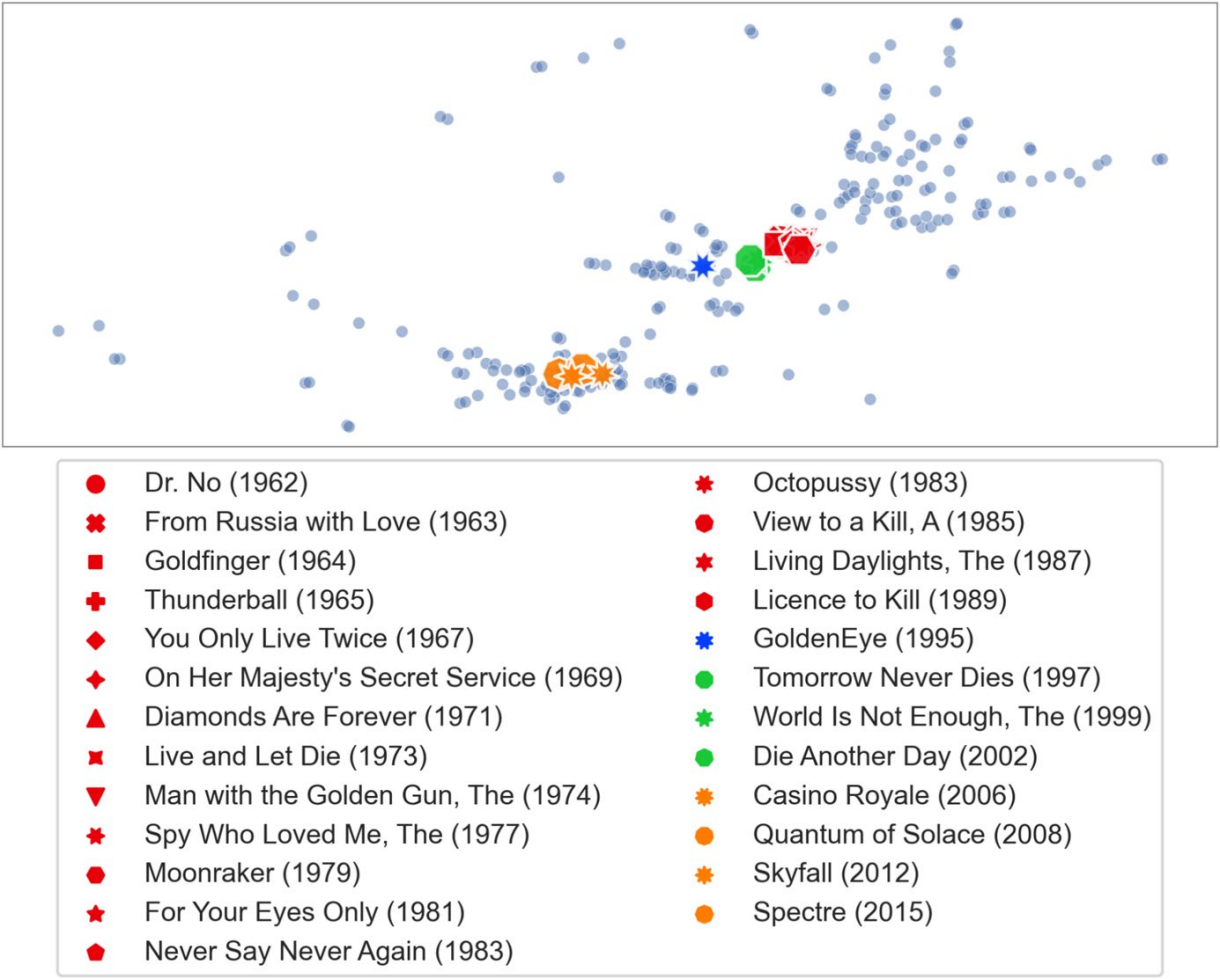
An embedding map of *James Bond* movies

**Fig. 16:**
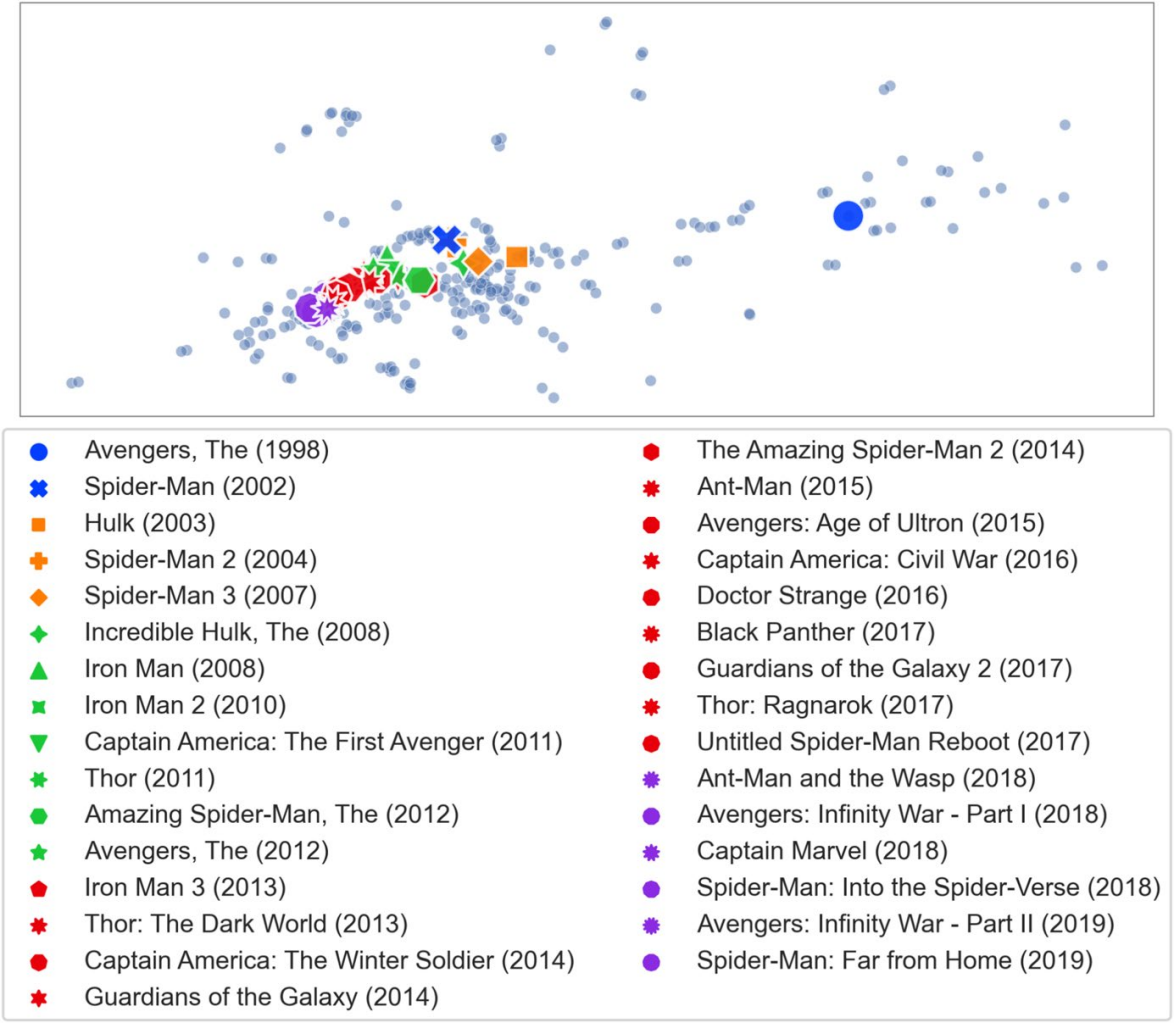
An embedding map of *Marvel* movies

**Visualizing clusters of movies.** Our dissimilarity measure can identify interesting features of the movies. In this experiment we consider clusters of movies that we used for TF-IDF callibration and show relations between them.

Star Trek. Let *A* consist of the fourteen *Star Trek* movies from MovieLens. We show the visualization of the extension of this set of movies in Fig. 13. The first ten movies, released between 1979 and 2002, are clustered closely together. These movies come from the original series and *The Next Generation* series (the transition between the two series was quite gentle, hence it is not surprising that the two groups are merged). The next cluster consists of the three movies from 2009, 2013, and 2016. These movies form a new, reboot series (so-called *Kelvin Timeline*). Finally, the 2015 movie is a fan film and does not belong to the official set. Altogether, we see that similar movies are grouped together even if they were released over a long period of time, whereas significant changes, such as making a reboot or shooting a fan film, are clearly separated.
Indiana Jones. Let us consider the series of four movies about Indiana Jones, an adventurer archaeologist. These movies are: *Indiana Jones and the Raiders of the Lost Ark (1981)*, *Indiana Jones and the Temple of Doom (1984)*, *Indiana Jones and the Last Crusade (1989)*, *Indiana Jones and the Kingdom of the Crystal Skull (2008)*. We show the visualization of the extension of this set of movies in Fig. 14. We see that the original trilogy, filmed in the 80 s, is placed close together, whereas the new movie from 2008 is quite far away. Indeed, as one may expect, reviving of the Indiana Jones franchise after nineteen years resulted in very mixed reactions from the fans. We find it quite interesting that such phenomena are visible in our dissimilarity measure.
James Bond. Movies from the *James Bond* series, depicted in Fig. 15, show similar effects as the *Star Trek* ones. The classic movies, shot before 1989 are clustered together. Then, after a six-year break (caused, in part, by some legal issues) and replacing Timothy Dalton by Pierce Brosnan in the role of James Bond (previously played by Sean Connery, George Lazenby and Roger Moore), we have the next cluster of four movies (*GoldenEye, 1995* is marked separately as it is viewed as the most successful of this group and stands out a bit). The final cluster of four movies marks a reboot of the series, and replacing Pierce Brosnan with Daniel Craig.
Marvel. Movies set in the *Marvel* universe, presented in Fig. 16, show a different phenomenon. Now, instead of a clear cluster, we have a progression of movies, grouped by the time when they were shot (movies are coloured in 5-year periods). This suggests that at some point people start watching *Marvel* movies, watch a few of them shot in some period of time, and then stop watching them.

The fact that our dissimilarity measure identifies such details as described above is an indication of its usefulness and quality.

### Committee diversity

One of the goals of our research is the control over the diversity of the exploratory search results. This section describes an experiment which measures the search diversity on the Movielens 25 M dataset when using *p*-HUV rules. The main conclusion is that, indeed, HUV rules can be used to easily control the results’ diversity simply by changing the *p* parameter.

**Measuring the results’ diversity.** To make it easier to describe the experiment, we call the movie that we search for the *query movie* or the *search movie*. We sometimes refer to the results of the search, that is, the winning committee, as the *winning movies*. In Sect. 4.3 we defined the $rank_a(b)$ function, which returns the rank of element *b* on the TF-IDF list when querying for element *a*. We refer to this rank as *b*’s TF-IDF rank (position) when searching for *a*, or simply as its rank if *a* is clear from the context. We can use this TF-IDF rank concept to measure the diversity of a winning committee when searching for a movie *a*: Given a committee, we take an average of the TF-IDF ranks of all the winning movies. We call this measurement a *query rank diversity* measure.

**Performing the experiment.** We sampled 1000 random movies from Movielens 25 M dataset and ran searches for each of them using 1-HUV, 2-HUV, 3-HUV. For each search we calculated the query rank diversity of the winning committee. For clarity, in most figures we display the results obtained with greedy algorithm only because the ones obtained with simulated annealing are similar.

**Fig. 17:**
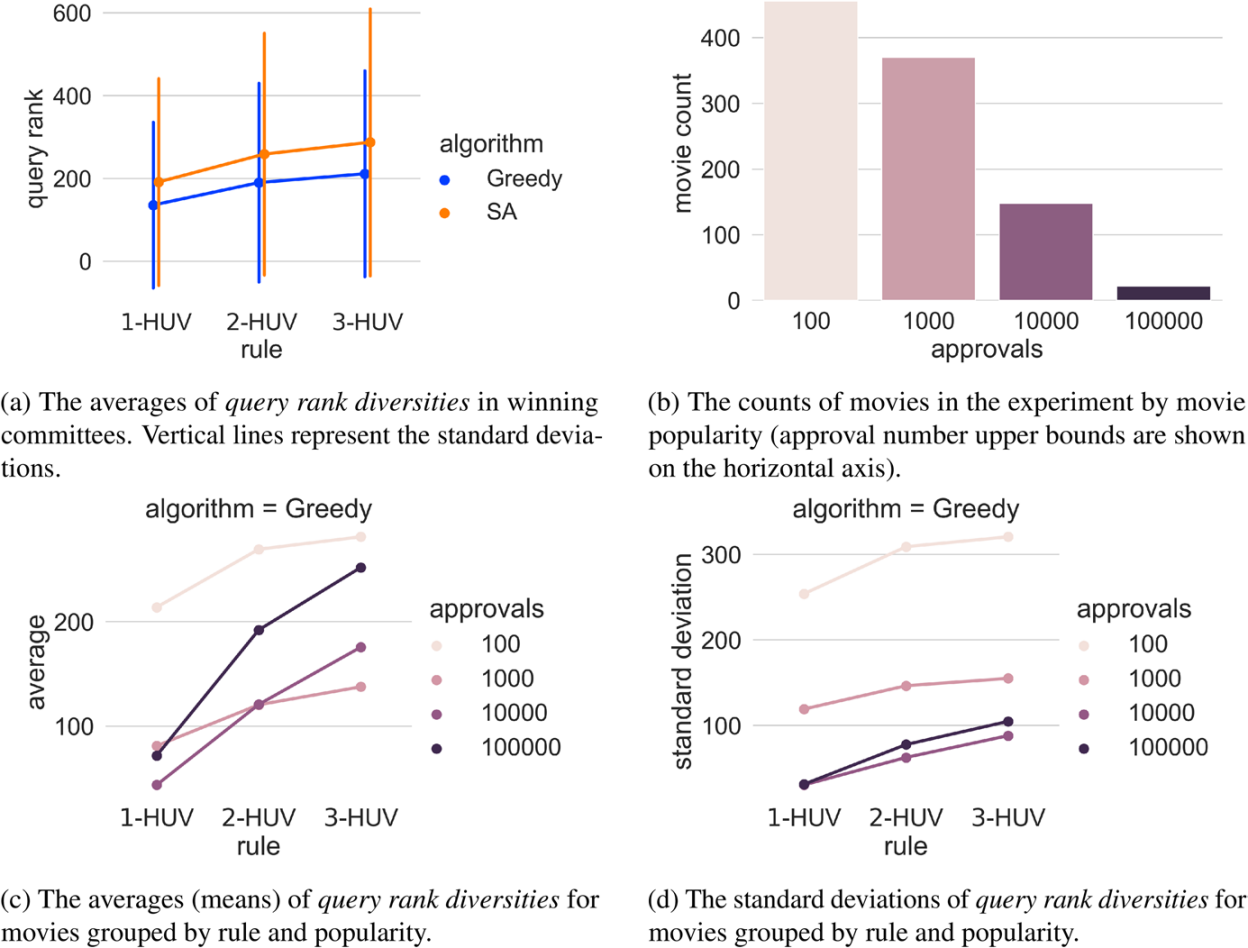
Results of the Committee Diversity Experiment, showing *query rank committee diversities* for 1000 randomly selected movies from the Movielens 25 M dataset

**Analysis.** We start with analysing query rank diversities and we draw their means together with their standard deviations in Fig. 17. We find that, as expected, the diversities of the committees increase with increasing *p* in *p*-HUV (see Fig. 17a). It is also interesting that the simulated annealing algorithm (SA) produces on average more diverse committees, but we point to the fact that this is dependent on the number of simulation steps used. We notice that the standard deviation of the movies (above 200) is high compared to the mean of around 100-300. To further investigate it we divide the committees in groups based on the popularity of the query movie measured by the number of approvals it gets. It turns out that the standard deviation is unusually high for less popular movies (the ones with low approval counts). We illustrate this fact in Fig. 17c, d, where we draw averages and standard deviations of the mean TF-IDF ranks of all committees bucketed by their query movie approval counts. In particular, we see that the least popular movies (which also appear in our data most frequently, see Fig. 17b) lead to highest standard deviation.

Nevertheless, the mean TF-IDF positions grow with growing *p* in *p*-HUV in each of the approval count groups. Thus, the results confirm our thesis that the diversity of the search results increases with growing parameter *p* in *p*-HUV rules.

## Conclusions and directions for future work

We have shown that multiwinner voting can be successfully used to build a system that helps searching for movies and that lets the users specify how strongly related should the proposed set of movies be to those he or she asks about. Interestingly, our approach can be used to identify nonobvious relations between the movies (as we have observed for the case of *Star Trek*, *Indiana Jones*, *James Bond*, and *Marvel* series of movies). Our system does not use any advanced tools for non-personalized recommendation systems and purely demonstrates that multiwinner voting is something that designers of such systems might want to consider. Yet, our system is a prototype and building it required a number of more-or-less arbitrary choices and decisions. We believe that most aspects of our work can be improved and we view our contribution largely as (a) offering a backbone on top of which further ideas can be tested, and (b) forming a baseline for evaluating future work. Below we describe possible directions for extending and improving our approach.

Foremost, our system is based on using multiwinner voting rules from the *p*-HUV family, but it is not at all clear that this is the best choice possible. Indeed, either there may be different families of OWA vectors that could give better outcomes, or—perhaps—voting rules based on completely different ideas. For example, it might be possible to use the growing theory of participatory budgeting elections, as the voting rules used there naturally accept candidates—or, in their language, projects—that have different utilities (Rey & Maly, 2023). In particular, different families of voting rules might be capable of achieving more appealing distributions of closely related, vaguely related, and not related committee members than our *p*-HUV rules achieve; see Sect. 4.2 and the discussion of the (*x*, *y*, *z*) vectors.

Another natural research direction is seeking and designing further heuristic algorithms for computing our rules. While we have focused on the classic greedy approach and simulated annealing, one could try various more advanced heuristic optimization techniques, such as, e.g., ant colony optimization (Dorigo & Stützle, 2019) or a number of other ideas. In particular, following this approach it might be possible to design algorithms that would choose committees of high quality that, nonetheless, would differ nontrivially with every execution. This would be quite valuable: If a user were not satisfied with search results, he or she might prefer to request results of a different run of the algorithm instead of extending the committee size and looking at items that, possibly, are too unrelated to his or her query. We believe that finding algorithms that output high-quality, but largely distinct committees at each run would be a very interesting research direction.^3^

A different direction for improving our approach would be to analyze further ways of deriving utilities in our elections. Specifically, we have used the classic TF-IDF heuristic and a fairly simple way of calibrating its parameters, so it would be natural to analyze other methods for distinguishing between movies that are relevant to the query and those that simply are popular overall. In particular, it would be very interesting to have an automated way of recognizing anomalies such as *Yamakasi* in Example 6 (a movie that by chance was liked by disproportionally many agents who enjoyed the query movie, but which otherwise was completely unrelated and unpopular). Another challenge would be to design automated algorithms for calibrating the $\gamma$ parameter of the TF-IDF heuristic, so that it could be tailored separately to each search. Indeed, in Sect. 4.1 we have seen that optimal $\gamma$ values may differ between queries (although we should also stress that the meaning of the term “optimal” here is not clear; in Sect. 4.1 we could rely on identifying a family of related movies, but in general such information would not be readily available).

Finally, there certainly is a lot of room for improvement regarding the dissimilarity measure between movies that we introduced in Sect. 4.3. In particular, as we have argued there, even if two movies are objectively very similar to each other, our measure can give different results depending on how many other movies are similar to them. This clearly is a flaw and circumventing it would be desirable.

## Data Availability

The code used to perform our experiments is available at https://github.com/Project-PRAGMA/Seach-Movies-Journal. This repository also includes information on how to obtain the MovieLens dataset of Harper and Konstan (2016), that we use (we cannot include it there due to its licence).

## Footnotes

1 Naturally, we could have used other criteria for forming $N_{ local }$, such as only considering voters that approve all members of *Q*. As we focus on singleton queries, this decision does not bear significant consequences.

2 We have also repeated the whole experiment for 50, 150, 200 and 500 global elections, but the results were essentially the same as for the case of 100.

3 We point out that while our simulated annealing algorithm might produce a different output at each run, different runs tend to generate outcomes that are quite similar. Hence, implementing the desiderata we just described would require something more than simply using a randomized optimization heuristic.
